# Insight into CMC-PVA-fHNTs Nanocomposite Hydrogel as an Advance Carrier for Cefadroxil Monohydrate: Fabrication and Characterization/Angiogenic Potential Analysis

**DOI:** 10.3390/gels10040235

**Published:** 2024-03-29

**Authors:** Saba Zia, Shahzad Maqsood Khan, Muhammad Taqi Zahid Butt, Nafisa Gull

**Affiliations:** 1 Institute of Polymer and Textile Engineering, University of the Punjab, Quaid-e-Azam Campus, Lahore 54590, Pakistan; nafisagull.ipte@pu.edu.pk; 2 Institute of Metallurgy and Materials Engineering, University of the Punjab, Quaid-e-Azam Campus, Lahore 54590, Pakistan; mtzbutt@hotmail.com

**Keywords:** sodium carboxymethyl cellulose, aminopropyltriethoxy silane, angiogenesis

## Abstract

Controlled drug delivery is a key strategy aimed at reducing both the frequency of therapeutic dosages and potential systemic side effects, particularly in the case of high drug concentrations. The nanocomposite hydrogel systems presented in this study were synthesized by combining carboxymethyl cellulose, polyvinyl alcohol, and (3-aminopropyl)triethoxysilane-functionalized halloysite nanotubes (fHNTs). This hydrogel system is a potential candidate for the controlled release of cefadroxil monohydrate. These hydrogels are analyzed by Fourier transform infrared spectroscopy, scanning electron microscopy, thermogravimetric analysis, and rheological measurements. Additionally, swelling properties, porosity, hydrophilicity, drug release, and in vitro and in vivo analyses were also evaluated. The observed trends in swelling and drug release demonstrated that the outcomes are dependent on the presence of fHNTs in the hydrogel matrix. Notably, fHNTs-loaded hydrogels displayed sustained drug release patterns. This innovative approach eliminates the need for traditional encapsulation and presents promising and translatable strategies for achieving more effective drug release.

## 1. Introduction

The effective distribution of drugs to treat multiple diseases has been the subject of biomedical research. Nanoparticles, liposomes, and hydrogels have been researched as potential drug delivery carriers. Among them, hydrogels are nontoxic, biodegradable and biocompatible and have excellent flexibility, elasticity, softness, and water absorption capabilities, which resulted in their extensive usage. Through interactions with physical or chemical crosslinking, they create a 3D network. This is typically accomplished using synthetic and natural polymers [1]. Controlling the drug encapsulation and delivery rate is achievable using a hydrogel, which proves beneficial as a drug delivery technology. Another benefit of hydrogels is based on the properties of the polymer, which can regulate the rate of drug delivery and encapsulation in response to variations in the external environment, like the pH or temperature. These advantages have led to the usage of hydrogels in the biomedical area for tissue engineering and drug delivery systems [2].

Polyvinyl alcohol (PVA) is water-soluble and partially crystalline with many hydroxyl groups that are easily able to create intermolecular hydrogen bonds [3]. Due to its great biocompatibility and mechanical attributes, it has been recognized as one of the essential materials utilized to synthesize hydrogels. PVA-based hydrogels can be created by crosslinking substances chemically and physically. Crosslinking agents such as glyoxal [4], glutaraldehyde [5], and borate [6] can be used to synthesize PVA-based hydrogels that have been chemically crosslinked. Repeated freeze–thaw cycles can cause physical crosslinking in PVA, producing crystallization and creating porous structures that connect the PVA network [7]. Due to the lack of the swelling ability of PVA-based hydrogels, PVA is typically mixed with other polymers that have a high capacity to absorb water, such as proteins, polysaccharides, etc. [8].

Polysaccharides, one of the ubiquitous natural polymers, have been extensively exploited as a hydrogel material due to their non-immunogenicity, biocompatibility, and functional adaptability. Polysaccharides can be produced from plants, animals, algae, and microbes [9]. Sodium carboxymethyl cellulose (Na-CMC) is one of the important polysaccharides. It has good film-forming properties and is water-soluble, inexpensive, and biodegradable [10]. It is a good material for drug delivery applications in the realm of hydrogels because it is highly changeable, nontoxic, and swellable [11]. Na-CMC-based hydrogels have been investigated extensively as medication carriers for water-soluble substances. Because of their biodegradability, biocompatibility, and solubility, these hydrogels may find use as drug delivery systems, sorbents, enzyme immobilizers, and wound-healing agents [12].

Cefadroxil monohydrate (Cef) is a first-generation cephalosporin and is a hydrophilic drug that fits in the range of broad-spectrum antibiotics. It is a widely used antibiotic for a broad range of bacterial infections of the skin, respiratory tract, and urinary tract infections (UTIs). However, to overcome the issue of a significant increase in staph infections, an efficient and biocompatible antibiotic delivery system is in urgent need [13].

The fascination of materials based on the mixture of PVA and Na-CMC is owing to the ease with which they can be synthesized and modified, as well as the variety of morphological characteristics they exhibit. It has been found that the crosslinking of Na-CMC with PVA significantly increases the thermal stability, mechanical strength, and flexibility. An appropriate crosslinking agent is employed to combine these polymers by creating entanglements in the polymer network, which enhances the film characteristics. The ability of silane molecules to create Si-O-Si bonds between the surface and silanol group allows for them to be used frequently as crosslinkers and adhesion promoters [14]. The organosilane (3-amonopropyl)triethoxysilane (APTS) can react with the OH groups of the polymers and create covalent bonds inside the hydrogel network, which improves the hydrogel stability and mechanical strength [15]. The use of nanoparticles has become a common technique to improve hydrogel characteristics. An existing study indicates that there is strong hydrogen bonding between the hydroxyl groups in PVA and the negative surface charge of halloysite nanotubes (HNTs). Therefore, this mixture can be easily stirred or combined with an ultrasonic technique to produce PVA/HNT composite films [16].

One such nanomaterial, HNTs, a naturally occurring mineral that resembles nanotubes, has drawn interest in uses for medication delivery. Halloysite’s distinctive tubular structure enables the encapsulation of substances within its inner lumen, providing stability and permitting prolonged release. HNTs improve the drug-loading capacity and offer a regulated drug release mechanism when incorporated into the hydrogel matrix, which increases the overall effectiveness of the drug delivery system [17]. However, there is no sufficient literature reported on the blend of PVA, Na-CMC, and APTS-modified HNTs. Therefore, the uniqueness of clay-based nanocomposite hydrogels resides in the creative method of employing clay nanofillers to synergistically synthesize, modify, and reinforce conventional polymeric matrices.

In this study, APTS was used as a crosslinking agent to synthesize hydrogels with Na-CMC/PVA and functionalized halloysite nanotubes (fHNTs) for regulated drug release. To determine the properties of the hydrogels, FTIR, SEM, and TGA analyses were performed. Additionally, the swelling, mechanical, and biodegradable characteristics of nanocomposite hydrogels were also examined.

## 2. Results and Discussions

### 2.1. FTIR Analysis

**Figure 1:**
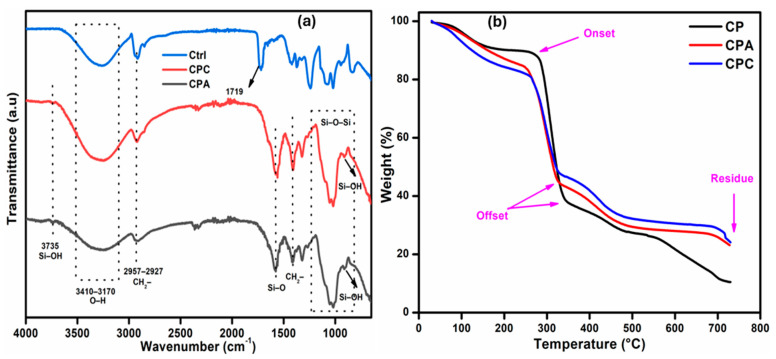
(**a**) FTIR spectra of prepared nanocomposite hydrogels. (**b**) TGA of prepared nanocomposite hydrogels.

FTIR analysis is used to assess the functional groups and proposed physical and chemical interactions among all the ingredients used to prepare the hydrogel. The FTIR spectra of the prepared hydrogels are shown in Figure 1a. The broad band at 3410–3170 cm^−1^ demonstrates the stretching vibration of the -OH groups present in the CMC, PVA, and fHNTs. This band is also overlapped with the -NH groups present in APTS [18]. The absorption bands at 2920 and 2850 cm^−1^ are successfully attributed to -CH asymmetric and symmetric vibrations, respectively [19]. The sharp band at 1580 cm^−1^ is linked to the acetyl group (C=O) of the CMC, and the band at 1422 cm^−1^ is due to the -CH bending vibration and at 1320 cm^−1^ it is due to the -CH wagging vibration [20]. The small bands at 1262 and 1020 cm^−1^ are owing to the acyclic and cyclic glycosidic bonds of CMC, respectively [21]. The bands in the array of 910–845 cm^−1^ are due to the Si-O-Si and Si-O-C groups of APTS [22]. On the basis of comparative spectra, it is clear that a peak shift or change in the intensity of the peaks has been observed, confirming the formation of polymer blend between Na-CMC and PVA. 

### 2.2. Thermogravimetric Analysis

TGA thermograms of the prepared samples are shown in Figure 1b. The curves present thermal stability of the systems in three different stages in a similar pattern. The first degradation stage involves the small weight loss (10–15%) from room temperature to 248 °C due to the loss of moisture, freeze water, and bound water. It is reported that weight loss of about 10% or more is recommended to achieve the onset of degradation. The second stage of degradation occurs in the range of 245–350 °C, which is a crucial stage because it indicates the partial hydrolysis of the PVA to yield polyene and degradation of the carboxylate groups of Na-CMC [21]. Third-stage degradation is associated with the dissociation of side chains and the backbone of the main polymers. Final-stage degradation, which is a plateau region, is ascribed to the decomposition of leftover carbonaceous material and ash formation [23].

It can be observed that fHNTs-loaded hydrogel shifts the onset to 263 °C, while it is 250 °C for the CPA samples. In addition, there is 24.5% residual weight, which is higher than the other samples, which shows the improved thermal stability of the fHNTs-loaded hydrogels. fHNTs act as a barrier in the hydrogels that is capable of wrapping the degraded materials inside its lumen when heated at a higher temperature and thereby shows improved thermal stability [24].

### 2.3. Swelling Ability of Hydrogels

#### 2.3.1. Swelling in Water

**Figure 2:**
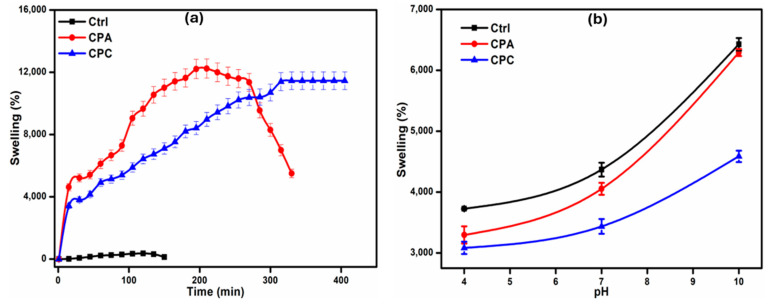
(**a**) Water swelling of prepared nanocomposite hydrogels. (**b**) pH swelling of prepared nanocomposite hydrogels.

The addition of fHNTs significantly affects the swelling of hydrogels as shown in Figure 2a. It is observed that with the addition of fHNTs, the swelling capacity of hydrogels decreases up to 19.43%. This decreasing trend is due to the blockage of the available pores of the hydrogel and the development of a very compact hydrogel network structure [25]. This trend is also associated with the dispersion of the fHNTs in a polymer matrix, which is attributed to the more coherent structure of the polymer with fHNTs, which ultimately fills the free spaces. HNTs are less hydrophilic than the polymer matrix, which also results in the reduction in the permeability of the hydrogels. With the addition of HNTs, they also act as an additional physical crosslinker, which reduces the water absorption capacity [26].

#### 2.3.2. Swelling in Buffer Solutions

The swelling of the hydrogels in buffer solutions of different pH is shown in Figure 2b. The swelling of the prepared hydrogels in the buffer solutions is mainly dependent upon the carboxylic and hydroxyl groups present in the constituent polymers. It can be observed that the swelling of the hydrogels in the buffer solutions is higher in the basic pH solutions rather than in the acidic solutions. At higher pH, the carboxylic groups of Na-CMC are deprotonated, which causes electrostatic repulsions between polymeric chains, which ultimately allows for more buffer solutions to penetrate in the porous network of hydrogels and cause more swelling [27]. But at acidic pH, the anionic groups are protonated, lowering the porosity of the hydrogels and subsequently reducing the swelling of the hydrogels. Both the polymers and fHNTs have abundant hydroxyl groups, which allow for hydrogen bonding with water molecules. The swelling behavior of hydrogels at lower pH is due to the hydrogen bonding between polymer chains and with the medium [19].

### 2.4. Porosity

Porosity is an important property of nanocomposite hydrogels to be used in drug release applications. Table 1 shows the porosity of the prepared samples. It can be observed that the control hydrogel sample shows the porosity of 78% due to the hydrophilic nature of both CMC and PVA, but this porosity percentage is decreased in the case of the CPA specimen. Due to the addition of crosslinker, the network structure becomes compact and dense due to which the porosity decreases [28]. The incorporation of fHNTs in the polymer network results in the clear decrease in porosity because fHNT particles might be stuck in the network, which ultimately decrease the porosity of the fHNTs-induced hydrogel [29]. The gel strength of the prepared nanocomposite hydrogels is also determined (Table 1), and it is found that it is 78% for the sample in which the crosslinker was used, but it is 74% for the CPC sample in which the fHNTs were induced in the hydrogel.

### 2.5. Hydrophilicity

The water-holding capacity of any material generally depends on the hydrophilic or hydrophobic nature of the components of that material. The hydrophilicity of the samples is measured using a water contact angle analysis. The contact angle of the prepared hydrogel samples (control, CPA, and CPC) is given in Table 1. The contact angle of all the hydrogel samples is observed to be less than 90, which means that all the samples are hydrophilic in nature. The hydrophilicity is observed to be decreased with the addition of APTS and further with the addition of fHNTs. Un-crosslinked polymer blends have usually been more hydrophilic than crosslinked ones [30]. The further addition of fHNTs in the crosslinked polymer blend shows less hydrophilicity owing to the more compact network because clay particles diffuse into the pores of the hydrogels. Furthermore, fHNTs are more hydrophobic than CMC and PVA, which is also the reason for a higher contact angle [31].

### 2.6. Scanning Electron Microscopy

**Figure 3:**
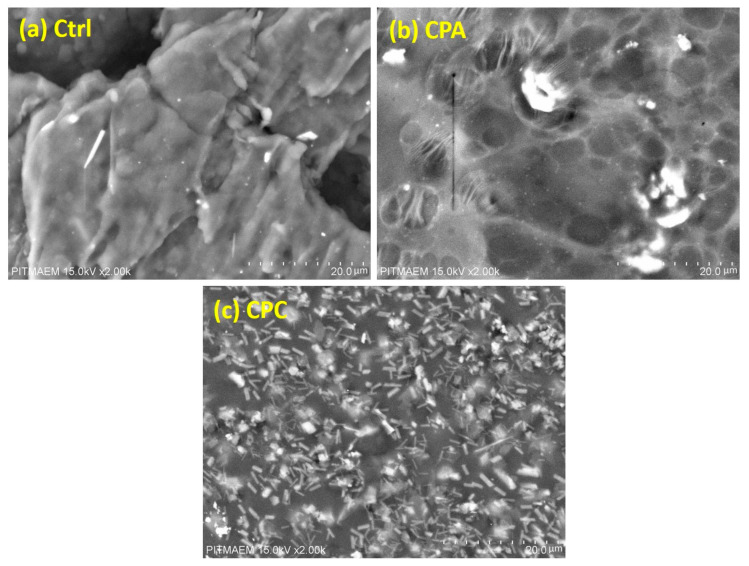
SEM micrographs of prepared nanocomposite hydrogels: (**a**) Ctrl, (**b**) CPA, and (**c**) CPC.

Figure 3 shows the SEM micrographs of the prepared control, crosslinked, and clay-induced hydrogels. The control hydrogel sample (Figure 3a) shows the porous network structure with interconnected micropores, which can absorb a large amount of water and other physiological fluids, which is confirmed by the water and pH swelling of hydrogels [32]. The micrographs (Figure 3b) show the network structure consisting of mesh, which provides the required hydrophilicity. In the fHNTs-induced hydrogel (Figure 3c), it shows a neater mesh, which is due to the crosslinking of the modified HNTs with polymer chains. Overall, it is clear that micrographs of hydrogels with a compact mesh-like network structure show less swelling ability [33].

### 2.7. Rheological Analysis

#### 2.7.1. Steady-State Flow Behavior/Thixotropic Behavior 

**Figure 4:**
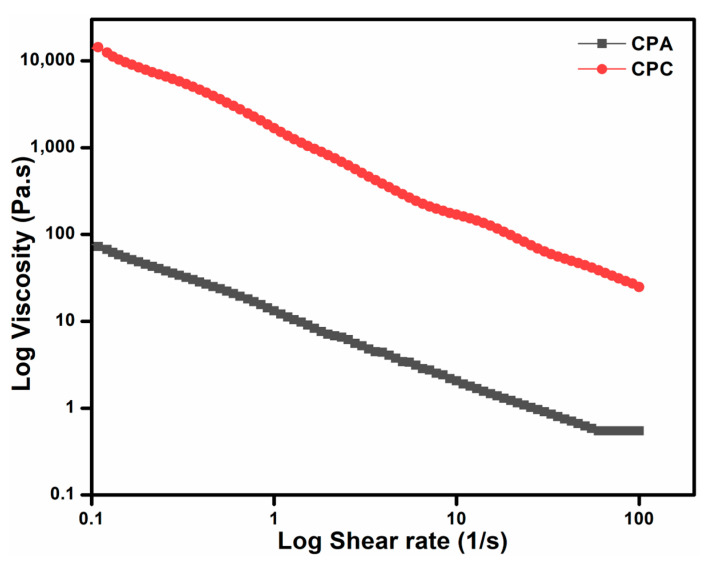
Shear rate vs. viscosity of prepared hydrogel.

The rheological trends of the shear stress and shear rate are presented in Figure 4. There is a decline in the shear viscosity with the increasing rate. The power-law model further confirms this shear-thinning nature of the flow curves. The equation of power law is given as (9):

(1) $$\tau =K\cdot {\dot{\Upsilon }}^{n}$$

where n is the power-law index, *τ* is the viscosity, $\dot{\Upsilon }$ is the shear rate, and *K* is the consistency index. The power-law-fitted parameters and values of the correlation coefficient (R^2^) are summarized in Table 2. The R^2^ values characterize the goodness of fit of the power-law model, and these values are ≥0.95; this shows that the model is a good fit and the viscosity behavior of both gels is within the given shear rate range. Moreover, the power-law index has values lower than unity that also confirms the shear-thinning behavior, which is an important property for drug release applications [34].

#### 2.7.2. Frequency Sweep Test

**Figure 5:**
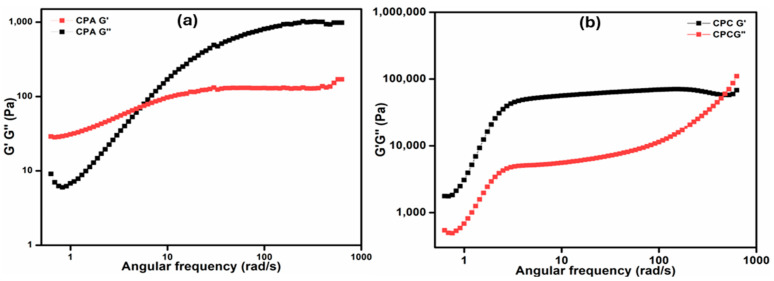
Angular frequency vs. G’ and G″ of (**a**) CPA hydrogel and (**b**) CPC hydrogels.

The frequency sweep test is used to check the supremacy of the elastic modulus over the viscous modulus (Figure 5a,b). The range of frequency that was employed in the test ranged from 0.01 to 100 Hz with the constant strain of 5%. From the rheograms, the crossover points for the CPA and CPC samples are observed at 3.5 Hz (33 rad s^−1^) and 75 Hz (480 rad s^−1^), respectively. The crossover points of the CPC go above when compared to the CPA that broken earlier during the test as observed in the results. Until the crossover point is reached, the elastic response is more dominating than the viscous response, which is the needed property for the tested nanocomposite hydrogels [35]. The angular frequency vs. G’ and G″ represent the sol–gel transition, which can be observed in CPC after 50 Hz (364 rad s^−1^), whereas CPA attains it after 10 Hz (60 rad s^−1^). A gradual increase in the loss factor can be seen before the sol–gel transition. This happens due to the increase in frequency; the crosslinked structures of the hybrid hydrogels start to lose their elastic nature.

#### 2.7.3. Strain Sweep Test

**Figure 6:**
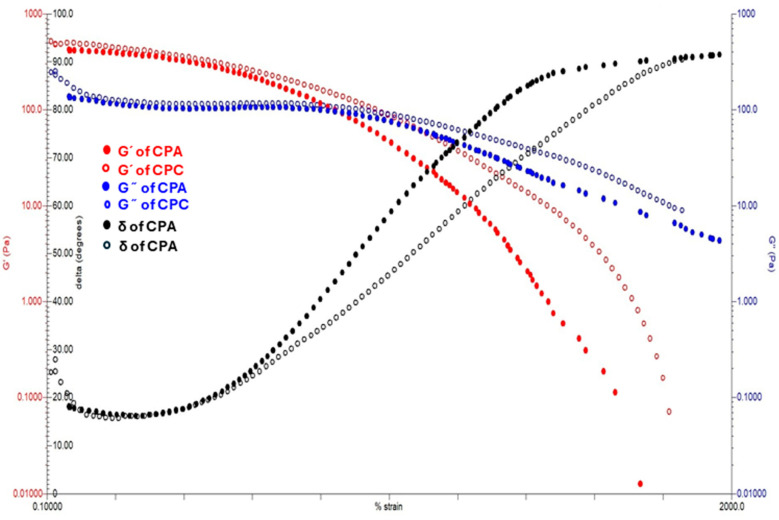
Storage modulus, loss modulus, and tan δ of prepared hydrogels.

These crosslinked polysaccharide-repeating units have a strong, long backbone chain and high molecular weight, which resist the deformation at small and applied shear rates [36]. So, a stable declining trend can be noticed in CPC as compared to CPA in Figure 6. Further, this decline is stiffer for CPC than CPA. Subsequently, this trend also shows that the viscosity of CPC is always greater than CPA. The viscoelastic properties of both hydrogels are also analyzed by the strain sweep test in which the % strain varied from 0.1 to 1000%. The rheogram curves clearly indicate the domination of the storage modulus over the loss modulus. The linear viscoelastic region of CPA is less than CPC, which indicates the increased crosslinks of CPC rather than CPA, which has shifted the plateau towards higher viscosities.

#### 2.7.4. Rheological Models

The noteworthy terms of the above Equations (3)–(6) are specified as follows: the shear rate *ϔ* (s^−1^), plastic viscosity η (Pa.s), shear stress *τ* (Pa), yield stress *τ*_0_ (Pa) related with the crucial point of stress that is applied to determine the start of the hydrogel flow, K associated with the viscosity of hydrogels is the consistency index (Pa.s^n^), and the flow behavior index n (dimensionless) related to the non-Newtonian or Newtonian character (n < 1 for a non-Newtonian pseudoplastic system, n > 1 for a non-Newtonian dilatant system, and n = 1 for a Newtonian system) [37].

The determination (R^2^) values in the (Table 2) are used as an indicator for the selection of the hydrogel that is the best fitted one for the forward flow profiles.

The values of R^2^ that are specific to the power-law/Ostwald–deWaele, Herschel–Bulkley, Bingham, Casson and Steiger/Ory models, presented in Table 2, indicate that the finest obtained are the Herschel–Bulkley, power-law, and Steiger/Ory models for all the prepared hydrogels CPA and CPC, in this case, with the R^2^ ranging between 0.9958 and 0.9916 at 37 °C, respectively. The descriptors specific to these models are listed in Table 3 for both hydrogel (CPA and CPC) systems tested at a temperature of 37 °C. The calculated parameters of all the above models are in Table 3.

The pseudoplasticity of the hydrogels can also be stated through the curves of the viscosity vs. shear rate, presented in Figure 4.

### 2.8. Antibacterial Properties

Antibacterial property is one of the important properties for hydrogels for their use in biomedical applications. In this study, the antibacterial activity against Gram-positive and Gram-negative bacteria is evaluated and the measured inhibition zone is shown in Table 4. The antibacterial activity of the control sample is low as its inhibition zone is observed as 4.84 mm, but this zone is enhanced up to 7.52 mm for CPA and further increased up to 11.27 mm for CPC. The reason for larger inhibition zone in the case of the CPC sample is that the outer surface of HNTs is negatively charged, which will interact with the cell wall of *S. aureus* and will destroy it, which ultimately shows the larger inhibition zone [38]. Overall, the antibacterial effect of the prepared hydrogels against *S. aureus* is greater than *E. coli*. The reason is that the structure of the cell wall with a layer of peptidoglycan between the cytoplasmic membrane and outer membrane of Gram-negative bacteria is more complex than that of Gram-positive bacteria [39]. Antibacterial analysis shows that the prepared hydrogels are potentially fit for use in biomedical applications. 

### 2.9. Cytotoxicity

Brine shrimp cytotoxicity testing is an inexpensive, rapid, and simple method to evaluate the toxicity of hydrogels used for biomedical application. Table 4 shows the percent mortality of the prepared hydrogels. It can be observed that all the samples show insignificant values of mortality. There is a nominal mortality, which might be due to two reasons: firstly, due to the toxic nature of the used chemicals and, secondly, due to the development of a viscous layer of the material solution around the gills of the nauplii. In this case, all the used chemicals are nontoxic and FDA-approved so the second one may be the reason for the small values of mortality [40]. Overall, the results show the fitness of the materials used in biomedical applications. 

### 2.10. Drug Release Analysis

**Figure 7:**
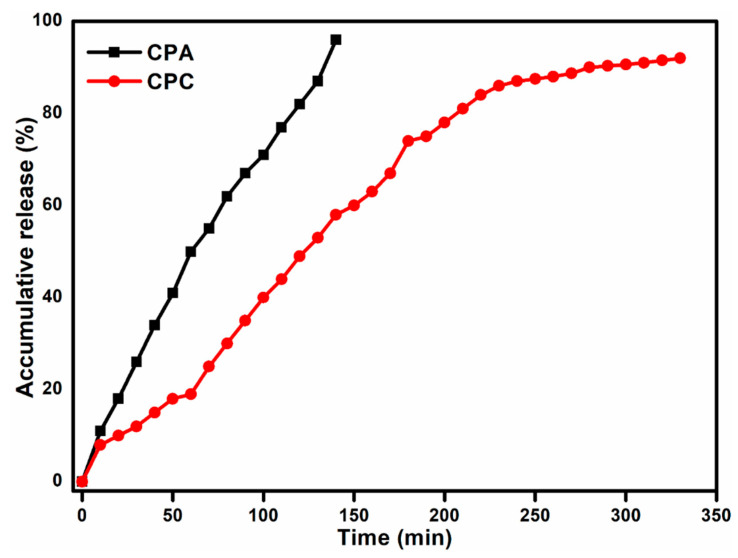
Drug release behavior of prepared hydrogel.

Figure 7 shows the accumulative drug release profile of the CPA and CPC hydrogel samples. It can be observed that in the CPA hydrogel, almost the whole drug is released in burst manner, but in the clay-loaded hydrogel, i.e., CPC, the drug is released in a controlled way (almost 95% release in 24 h). During the drying of hydrogel films, most of the drug moves toward the surface of the films along with the solvent, resulting in the large number of molecules concentrated near the surface of the film, which causes a burst release. But with the addition of fHNTs, drug molecules undergo physical and chemical interactions with polymers and fHNTs, which cause the entrapment of the drug within the polymer network and prevent the movement of drug molecules toward the surface and subsequently control the release of the drug from the polymer matrix [20].

### 2.11. In Vivo Analysis

#### 2.11.1. Angiogenic Potential of Hydrogels 

**Figure 8:**
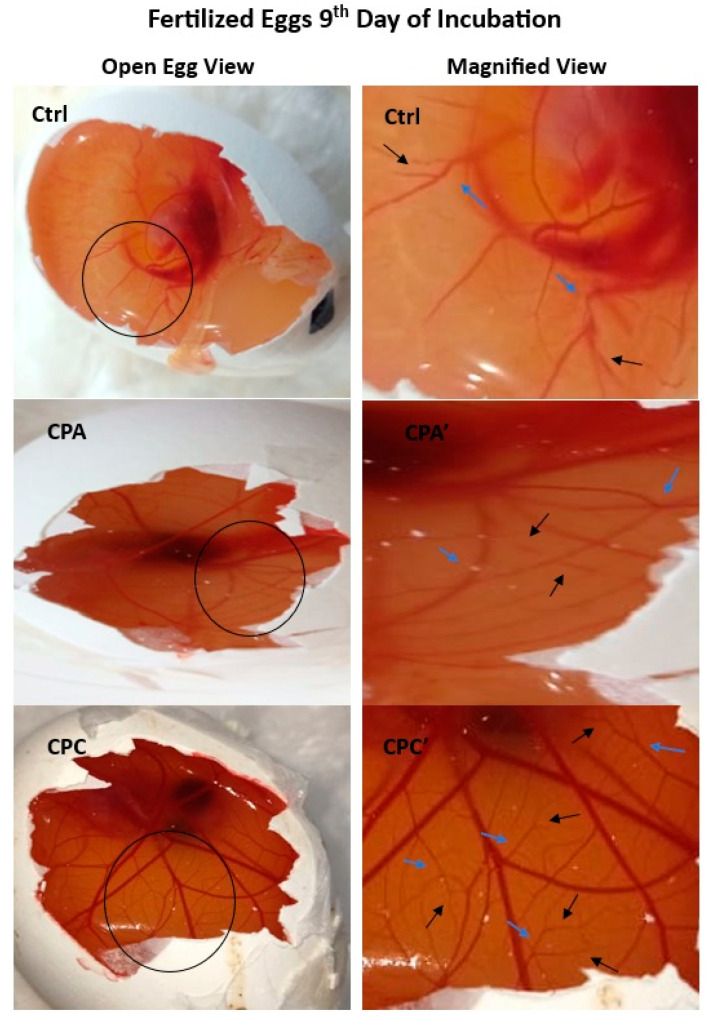
Neovasculogenic and angiogenic potential of Ctrl, CPA, and CPC during chick development at 9th day.

The prepared nanocomposite hydrogel samples are studied with the Chorioallantoic membrane (CAM) assay for angiogenesis and vasculogenesis on the 9th day of their incubation (Figure 8). Observing the vasculature layout, it is noticed that the CPA and CPC hydrogels response is in the form of remarkable blood vessel formation as compared to the Ctrl sample. This Figure is marked with arrows in the figure (blue arrows indicating secondery and black arrows indicating tertairy blood vessels). A regular angiogenic blood vessel pattern is visible in the control sample. fHNTs have been found biocompatible; this means they are well borne by the living cells and do not promote significant harmful reactions. Accordingly, when combined with a biocompatible hydrogel matrix, fHNTs produce more favorable conditions for angiogenesis. fHNTs have good adhesion and migration characteristics, which are very crucial for angiogenesis processes. fHNTs provide a suitable and larger surface area for endothelial cell movement and attachment. Moreover, fHNTs prove their low toxicity which made them safe for control drug release applications. This property is also essential when assessing biological and hydrogel safety in an angiogenic assessment. Thus, CPA and CPC are ensuring materials for the acceleration of neovascularization. Among both samples, CPC performs even better because fHNTs have the enhanced biological characteristics of hydrogels [41,42].

#### 2.11.2. Quantification of Blood Vessels

**Figure 9:**
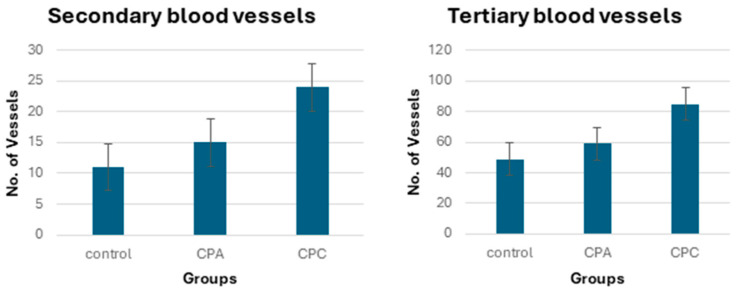
Neovasculogenic and angiogenic potential of ctrl, CPA, and CPC during chick development at 9th day.

The blood vessels of the chick embryos are analyzed with the help of high-definition images recorded per unit of area. The data obtained from this analysis (Figure 9) depicts a significant increase in the thickness and number of blood vessels counted with the incorporation of APTS in the CPA. However, better result than the control and CPA is shown by the CPC. The number of average blood vessels (secondary and tertiary) of the control (experimental control and reference control), CPA, and CPC are shown in Figure 8 and Figure 9. The CPA and CPC both show more angiogenic potential than the control with no visible toxic effects. This might be because of the APTS that possesses antioxidative characteristics due to (-NH_2_) [43]. 

#### 2.11.3. Toxicity Testing Morphological and Morphometric Analyses

**Figure 10:**
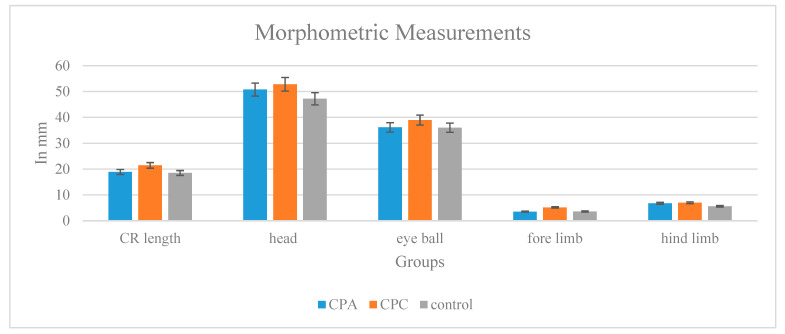
Morphometric measurements of control, CPA, and CPC.

The gross studies of the hydrogel-treated chick embryos recovered on the 9th day shows normal growth features. Considering the average morphometric measurements of the body parts of the recovered embryos, there is not much difference between the control, CPA, and CPC (Figure 10). The recorded body weights of the control, CPA, and CPC-treated embryos (control—600 mg, CPA—697 mg, and CPC—830 mg) also favor the growth factors regarding the CPC hydrogel as compared to the CPA and control. 

#### 2.11.4. Amniotic Fluid Analysis 

Table 5 presents the amniotic fluid biochemical markers of the chick embryos for the evaluation of their liver and kidney function of the control, CPA, and CPC hydrogel-treated embryos. There is not much significant difference observed in the level of enzymes like the AST, ALT, and ALP of the CPA and CPC in comparison to the control. Similarly, the results of the RFT and LFT display that these hybrid hydrogels are nontoxic. However, minor raised levels observed for CPA may be because of the concentrations of APTS [44]. 

## 3. Conclusions

Recent advancements in the development of nanocomposite hydrogels, specifically those based on fHNTs-loaded hydrogels, have fueled progress in the biomedical field, particularly in the domain of controlled drug release systems. This crosslinked hydrogel has the potential to serve as an effective drug delivery system. These biodegradable hydrogels exhibit their maximum swelling capacity in various environments, including distilled water and acidic buffer solutions. The evaluation encompasses antimicrobial properties against bacterial strains, and cytotoxicity assessments using brine shrimp lethality assays, and angiogenic potential through CAM assay, respectively, showing nontoxic and supportive behavior. Furthermore, by the loading of fHNTs, these hydrogels acquire remarkable thermal stability and structural properties, attributed to the formation of a crosslinked network structure exhibited in rheological measurements by shear thinning, and linear viscoelastic regions improve with fHNTs and G′ > G″. Parameters such as porosity and hydrophilicity are also examined. These characteristics endorse the utility of the prepared hydrogels for controlled release systems. These hydrogels exhibit a tunable controlled release of Cef, achieved by the addition of fHNTs.

## 4. Materials and Methods

### 4.1. Materials

The sodium carboxymethylcellulose (Na-CMC) (Commercial grade, Lahore, Pakistan) was obtained from Panreac Applichem, Darmstadt, Germany. The polyvinyl alcohol (PVA) of MW 30,000–70,000 g/mol, 87–90% hydrolyzed, was purchased from Sigma Aldrich, St. Louis, MI, USA, the halloysite nano clay was purchased from Sigma Aldrich, and the (3-aminopropyl)triethoxysilane (APTS), ethanol, potassium dihydrogen phosphate, sodium dihydrogen phosphate, and potassium chloride were purchased from Merck, Germany. All the chemicals were of analytical grade and used without any further treatment.

### 4.2. Method

#### 4.2.1. Functionalization of HNTs

The APTS solution was prepared as ethanol/water in 6:4 for functionalization. A total of 0.6 g of HNTs was added into the prepared mixture. This suspension was firstly mixed for 2 h using a magnetic stirrer and then sonicated for 30 min to obtain homogeneous dispersion. The treated HNTs were then dried in an oven at 100 °C for the complete removal of ethanol, thus forming fHNTs [45]. 

#### 4.2.2. Fabrication of Nanocomposite Hydrogel

The Na-CMC (0.5 g) was dissolved in 100 mL of distilled water at room temperature and PVA (0.5 g) was dissolved in 100 mL of water at 90 °C. To prepare the control sample, both solutions were blended at 50 °C for 3 h. This blend was cast into a petri dish and dried in an oven at 60 °C. To prepare the APTS crosslinked sample, 50 μL of APTS was mixed in 5 mL of ethanol and added in the above prepared blend, mixed for further 3 h, and then poured in a petri dish and dried in an oven at 60 °C. For the preparation of the fHNTs-loaded hydrogels (CPC), 0.05 g of fHNTs was dispersed separately in ultrapure water (100 mL) by magnetic stirring for 30 min and ultrasonicated at 700 W for 30 min. Subsequently, the Na-CMC and PVA well-blended mixture (1:1) was added into the above solution and continuously stirred overnight in the presence of fHNTs at room temperature. The mixed solution was poured in plastic petri dishes and dried in a drying oven at 60 °C. The codes of the different samples are presented in Table 6.

#### 4.2.3. Fabrication of Drug-Loaded Nanocomposite Hydrogel

To prepare the drug-loaded nanocomposite hydrogels sample, a first-generation cefadroxil monohydrate (Cef) antibiotic was used as a model drug. In the first step, 0.5 g of Na-CMC was dissolved in distilled water at room temperature, and 0.5 g of PVA was dissolved in 100 mL of water at 90 °C. Both solutions were blended for 2 h at 30 °C; 50 mg of the model drug powder was dissolved in 5 mL of water and added into the above prepared blend, and this solution was stirred for 1 h; and 50 μL of APTS was dissolved in 5 mL of ethanol and added dropwise in the above solution. This blend was magnetically stirred for further 4 h. The same procedure was followed for the preparation of fHNTs-loaded hydrogels (CPC); the drug solution was added dropwise in the prepared blend of Na-CMC/PVA/fHNTs. After that, the drug-loaded prepared blends of the samples of the crosslinked hydrogels and fHNTs-loaded hydrogels (CPA and CPC) were poured in the plastic petri dish and dried at 50 °C in a drying oven. After complete drying, the dried sample was peeled off from the petri dish and stored in a desiccator to avoid any moisture absorption.

### 4.3. Fourier Transform Infrared Spectroscopy

FTIR was performed using the IR Prestige 21, Shimadzu, Kyoto, Japan, and confirmed the presence of the functional groups and physical and chemical bonding in the samples. A scan rate of 20 scans per sample with a resolution of 6 cm^−1^ was used to analyze the samples in the wavenumber range of 4000 to 650 cm^−1^.

### 4.4. Swelling Experiments

#### 4.4.1. Swelling in Distilled Water

The pre-weighed samples were put in a sufficient amount of water for immersion. The swollen hydrogel samples were removed after 10 min interval, and the surface water was then dried with blotting paper. This procedure was continued until the consistent weight was reached. Equation (1) was used for calculating the swelling profile:

(2) $$Swelling\;\left(\%\right)=\frac{Ws-Wd}{Wd}\times 100$$

where *Ws* represents the swollen weight and *Wd* corresponds to the dried weight [46].

#### 4.4.2. Swelling in Buffer Solutions

The swelling profile of the prepared hydrogels was also studied in various buffer solutions. Standard buffer solutions of pH 4, 7, and 10 were used, obtained from Fisher scientific, and the above procedure was followed to assess the swelling. Equation (1) was used to measure the swelling of the samples following the corresponding equilibrium time.

### 4.5. Porosity

The solvent displacement method was applied for the porosity analysis. The dimensions of the hydrogel were measured using a digital vernier caliper (Fowler 6″/150 mm Pro-Max Electronic Caliper 54-200-777-1 (WESTport Corporation, West Islip, NY, USA)). From these readings, the volume was calculated. The displacement solvent was absolute ethanol. The weighted hydrogel films were logged in absolute ethanol so the ethanol would penetrate the sample pores. The excess ethanol was wiped, and the weight of the samples was recorded. The porosity (%) calculation was performed by following Equation (2):

(3) $$\mathrm{Porosity}\;(\%)=\frac{M_{2}-M_{1}}{\rho V}\times 100$$

M_1_ represents the dried weight, M_2_ is the weight of the hydrogel after ethanol submersion, V is the volume of the hydrogel, and ρ is the density of the ethanol.

The gel strength of the prepared samples was also determined using a soxhlet apparatus.

### 4.6. Hydrophilicity

The hydrophilic characteristics of the synthesized specimens were evaluated using contact angle measurements. The hydrogel samples were placed on microscopic slides by using a goniometer instrument (Kernco Instrument Co. Inc., El Paso, TX, USA), and the water–film contact was studied. With the aid of a syringe, a droplet of distilled water was gently applied on the surface of the sample, and the contact angles were recorded from both sides of the water droplets. The observed data were the ±SD of 20 recordings per sample [47]. 

### 4.7. TGA 

TGA was utilized to evaluate the thermal stability of the samples. The experiments were performed on SDT Q600 (simultaneous DSC/TGA) TA Instruments, 159 Lukens Dr, New Castle, DE, USA. The hydrogel specimens were heated from room temperature to 800 °C at the ramp rate of 20 °C/min in an inert environment with a nitrogen flow of 100 mL/min.

### 4.8. Scanning Electron Microscopy 

The hybrid hydrogels morphological measurements were examined under SEMFEI Nova Nano SEM 450 EDX-Oxford X-act, USA. The imaging of the hydrogels was improved by the application of a gold sputter coating.

### 4.9. Rheological Properties 

The parallel plate rheometer (AR 1500 EX, TA Instruments, New Castle, DE, USA) was outfitted with a 40 mm parallel plate for evaluating the dynamic viscoelastic properties of the prepared hydrogel systems. The gap between the measurement plate and peltier plate was fixed at 52 µm. The oscillation and flow processes were chosen for the measurements. The shear rate from 0.1 to 100 s^−1^ was used to evaluate the stable shear viscosity during the flow operation. A 5% strain was applied in the dynamic oscillatory flow measurements to ensure that the results were in the range of the linear viscoelastic region. The elastic/storage modulus (G′) and the viscous/loss modulus (G″) were calculated for a wide range of angular frequencies (0.682–682 rad/s). Additionally, the experiments using stresses ranging from 0.1 to 100% were performed at a static angular frequency of 6.283 rad/s, and a temperature of 37 °C was used for all the experiments. 

### 4.10. Rheological Models

The shear stress and shear rate relationship was furthermore investigated using different models of rheology: Ostwald–de Waele/power law (Equation (3), Herschel–Bulkley (Equation (4), Bingham (Equation (5), and Steiger/Ory and Casson (Equation (6) were employed in this study [48].

(4) $$\tau =K\cdot {\dot{\Upsilon }}^{n}$$

(5) $$\tau =\tau_{0}+K\cdot {\dot{\Upsilon }}^{n}$$

(6) $$\tau =\tau_{0}+\eta \cdot \dot{\Upsilon }\;$$

(7) $$\tau^{0.5}={\tau_{0}}^{0.5}+\eta^{0.5}\cdot {\dot{\Upsilon }}^{0.5}\;$$

The noteworthy terms of the above Equations (3)–(6) are specified as follows: shear rate *ϔ* (s^−1^), plastic viscosity η (Pa.s), shear stress *τ* (Pa), yield stress *τ*_0_ (Pa) related to the crucial point of stress that is applied to determine the start of hydrogel flow, K associated with the viscosity of the hydrogels, is the consistency index (Pa.s^n^), and the flow behavior index n (dimensionless) related to the non-Newtonian or Newtonian character (n < 1 for a non-Newtonian pseudoplastic system, n > 1 for a non-Newtonian dilatant system, and n = 1 for a Newtonian system) [49].

### 4.11. Bio-Assessment Tests

#### 4.11.1. Antimicrobial Activity

The antimicrobial activity of the hydrogels was investigated using the modified disc diffusion method. The LB plate surfaces were immunized with bacterial pathogens with sterilized cotton wads, diluting them afterwards with the 0.5 McFarland standard. Discs of 0.7 mm in diameter of the hydrogels were aseptically added onto the dish surfaces by means of tip-kindled pincers. These plates were then placed in incubators at the temperature of 30 °C for 24 h. Afterwards, the inhibition zones (mm) were measured by analyzing the disc diameter of each sample [26].

#### 4.11.2. In Vitro Cytotoxicity Analysis

An in vitro cytotoxicity investigation was carried out using the brine shrimp lethality bioassay. The brine shrimps were hatched in a sterile artificial seawater container with constant aeration at room temperature for 24 h. The mature nauplii were removed from the brighter part of the container and used as a bioassay in a microtiter plate with wells that were 1.8 cm in diameter and 2 cm deep. The sea water in each well measured 0.2 mL in volume. The immature larvae were counted, and the hydrogel samples were obtained in triplicate in the active nauplii wells. This well plate was kept at room temperature and kept in the dark for 24 h. The surviving nauplii were counted and viewed using an optical microscope (GXM, XPL33230 GT vision, Heverhill, UK) using the following Equation (7):

(8) $$M\left(\%\right)=\frac{A-B-N}{G-N}\times 100$$

while *M* represents the proportion of dead nauplii after 24 h, *A* represents the actual number of dead nauplii, and *B* represents the typical number of dead nauplii after 25 h. The letters *N* and *G*, stand for the number of dead nauplii present prior to the test start and the overall number of nauplii, respectively [50].

#### 4.11.3. In Vitro Drug Release Profile

The drug-loaded hydrogel film was placed in a beaker containing 100 mL of PBS solution and heated to 37 °C. Next, 5 mL aliquots of this solution were taken after every 10 min for 3 h, and then 5 mL of fresh PBS solution in the same volume was added. Each sample absorbance was measured at 535 nm using a UV–visible Spectrophotometer, Double Beam, Perkin Elmer, Model Lambda 25, USA, to calculate the percentage of drug release. Equation (8) was used to calculate the *cumulative drug release* from the prepared hydrogel:

(9) $$Cumulative\;drug\;release\;\left(\%\right)=\frac{Amount\;of\;drug\;released}{Total\;amount\;of\;drug\;in\;the\;hydrogel}\times 100$$

#### 4.11.4. Chorioallantoic Membrane Assay for the Assessment of Angiogenic Properties of Hydrogels and Toxico-Pathological Analyses

The analysis procedure includes eggs (fertilized and fresh) *Gallus domestics* of the white leghorn class; these fresh eggs were bought from the Veterinary Research Institute, Lahore, Pakistan (VRI). The whole 175 eggs were firstly disinfected very carefully using 20% alcohol wads. All the eggs were incubated for the period of 9 days at the temperature of 37 ± 1 °C in moistened digital incubators, which were self-rotatory for the egg’s positions. The eggs were additionally allocated into two handling groups: control, CPA, and CPC hydrogels in the first assembly; the other ones were left untouched, taking them for standards as the control in the second assembly. The incubated eggs were candled to confirm their fecundity on the fourth day of observation. The fertilized eggs’ embryotic movements with visible patterns of blood vessels could be detected and then a 1 cm^2^ square opening was marked open on the shells of the fertilized eggs, marginally at the side of the embryo, on the chorioallantoic membrane. The hydrogels after sanitation were positioned on the CAM; after that, the eggs were sealed again by sterilized parafilm tape while waiting for the 9th day of incubation [51].

#### 4.11.5. Digital Imaging, Amniotic Fluid Sampling, and Embryos Recovery

The incubated eggs were examined cautiously on the 9th day of incubation. The vascular plexuses of the embryos were digitally photographed to detect the angiogenic response of the hydrogel systems along with the quantification of the blood vessels. This quantification was performed by using ImageJ 1.8.0 through the circular marking of the uniform dense vasculature zones in triplicate. The samples of the amniotic fluids were drawn from the embryo’s amniotic sac with the help of a 21-gauge syringe and the embryos were recuperated for the gross morphological assessments.

#### 4.11.6. Enzyme Assays

The collected amniotic fluid samples were placed in a centrifuge and were centrifuged at 3000 rpm for 15 min; the supernatants were saved at −80 °C until they were assessed. The enzyme alkaline phosphatase (ALP), aspartate aminotransferase (AST), alanine aminotransferases (ALT), and bilirubin were found for the liver functioning. For the renal function assessment, the urea levels and creatinine were measured from the supernatant recovered for the liver tests by means of Bayer’s commercial kits; the kit protocols were followed over completely automatic chemistry analyzers.

#### 4.11.7. Morphological Observations 

The embryos that were retrieved were fixed in Bouin’s fixative for 48 h at a temperature of 25 ± 2 °C. After 48 h, photographic measurements of the embryos were taken and then the embryos were transferred to a 70% ethanol solution.

### 4.12. Statistical Analysis

After each swelling experiment was run three times, the experimental data were reported as the mean ± SD by the origin Pro8 software version. The Student *t*-test was used to compare the statistical significance. P values under 0.05 were regarded as statistically significant.

## Figures and Tables

**Table 1:** Porosity and hydrophilicity of prepared hydrogels.

Sample Codes	Porosity (%)	Hydrophilicity (°)	Gel Strength (%)
Ctrl	78	69	67
CPA	69	65	78
CPC	55	62	74

**Table 2:** Power-law parameters and determination coefficient (R^2^) values calculated from different rheological models acquired relative to rheograms for nanocomposite hydrogels tested at 37 °C.

Sample Code	Power Law			Ostwald–De	Herschel–Bulkley	Bingham	Casson	Steiger/ Ory
	m	n	R^2^	R^2^	R^2^	R^2^	R^2^	R^2^
CPA	14.4575	0.28	0.9916	0.9958	0.9958	0.8507	0.9853	0.8651
CPC	1644	0.47	0.9571	0.9134	0.5330	0.7899	0.7398	0.9916

**Table 3:** Herschel–Bulkley and Steiger/Ory parameters acquired relative to rheograms for nanocomposite hydrogels tested at 37 °C.

Sample Codes	Herschel–Bulkley Parameters			Steiger/Ory Parameters	
	*τ*_0_ (Pa)	*K* (Pa.s^n^)	n	K1	K2
CPA	0.5	14.456	0.2823	0.0139	0.28
CPC	1.2	1644.6	0.0591	1644	0.47

**Table 4:** Antimicrobial activity and mortality of prepared hydrogels.

Sample Code	Inhibition Zone (mm)		Mortality (%)
	*E. coli*	*S. aureus*	
Ctrl	2.56	4.84	4.58
CPA	3.27	7.52	6.41
CPC	6.59	11.27	7.89

**Table 5:** Biochemical markers of amniotic fluid in chick embryo following hydrogel treatment in comparison with control.

Parameters	Untreated	Treated Groups (Mean ± S.E)	
	Control	CPA	CPC
Bilirubin (mg/dL)	0.76 ± 0.08 ^a^	0.23 ± 0.08 ^b^	0.23 ± 0.08 ^b^
ALP (U/L)	113.00 ± 1.15 ^a^	143.00 ± 2.15 ^c^	124.00 ± 2.07 ^b^
ALT (U/L)	7.66 ± 1.20 ^a^	10.00 ± 1.15 ^b^	8.33 ± 0.88 ^b^
AST (U/L)	9.33 ± 0.88 ^a^	13.00 ± 1.15 ^b^	10.33 ± 0.88 ^c^
AST (U/L)	9.33 ± 0.88 ^a^	13.00 ± 1.15 ^b^	10.33 ± 0.88 ^c^
Creatinine (mg/dL)	0.76 ± 0.08 ^b^	0.56 ± 0.12 ^b^	0.53 ± 0.88 ^b^

Different alphabets (a, b and c in superscripts) on values show significant difference among groups. Numerical values are expressed as (Mean ± S.E); groups with different alphabets showed significant difference (*p* ≤ 0.05) from each other.

**Table 6:** Sample codes of prepared nanocomposite hydrogel samples.

Sample Codes	Na-CMC (g)	PVA (g)	APTS (μL)	fHNTs (g)
Ctrl	0.5	0.5	0	0
CPA	0.5	0.5	50	0
CPC	0.5	0.5	0	0.05

## Data Availability

The data presented in this study are available on request from the corresponding author.
